# Diagnosis and treatment of Paget’s disease of bone: position paper from the Italian Society of Osteoporosis, Mineral Metabolism and Skeletal Diseases (SIOMMMS)

**DOI:** 10.1007/s40618-024-02318-1

**Published:** 2024-03-15

**Authors:** D. Rendina, A. Falchetti, D. Diacinti, F. Bertoldo, D. Merlotti, S. Giannini, L. Cianferotti, G. Girasole, M. Di Monaco, S. Gonnelli, N. Malavolta, S. Minisola, F. Vescini, M. Rossini, B. Frediani, I. Chiodini, F. Asciutti, L. Gennari

**Affiliations:** 1 Department of Clinical Medicine and Surgery, University of Naples “Federico II”, 80138 Naples, Italy; 2 Department of Medical Biotechnology and Translational Medicine, University of Milan, 20122 Milan, Italy; 3 Department of Radiological Sciences, Oncology and Pathology, Sapienza University of Rome, Viale Regina Elena 324, 00161 Rome, Italy; 4 Emergency Medicine, Department of Medicine, University of Verona, 37129 Verona, Italy; 5 Department of Medical Sciences, Azienda Ospedaliera Universitaria Senese, 53100 Siena, Italy; 6 Clinica Medica 1, Department of Medicine, University of Padova, 35122 Padua, Italy; 7 Bone Metabolic Diseases Unit, Department of Experimental, Clinical and Biomedical Sciences, University of Florence, 50121 Florence, Italy; 8 Rheumatology Department, La Colletta” Hospital, ASL 3 Genovese, 16011 Arenzano, Italy; 9 Osteoporosis Research Center, Fondazione Opera San Camillo, Presidio Di Torino, 10131 Turin, Italy; 10 Department of Medicine, Surgery and Neurosciences, University of Siena, 53100 Siena, Italy; 11 Casa Di Cura Madre Fortunata Toniolo, and Centri Medici Dyadea, 40141 Bologna, Italy; 12 U.O.C. Medicina Interna A, Malattie Metaboliche Dell’Osso Ambulatorio Osteoporosi E Osteopatie Fragilizzanti, Sapienza University of Rome, 00185 Rome, Italy; 13 Unit of Endocrinology and Metabolism, University-Hospital S. M. Misericordia, Udine, Italy; 14 Rheumatology Unit, University of Verona, Policlinico GB Rossi, 37134 Verona, Italy; 15 Department of Biotechnology and Translational Medicine, University of Milan, 20122 Milan, Italy; 16 Ospedale Niguarda Cà Granda, Piazza Ospedale Maggiore 3, 20161 Milan, Italy; 17 Associazione Italiana Malati Osteodistrofia Di Paget, Siena, Italy

**Keywords:** Paget’s disease of bone, Metabolic bone diseases, Bone deformities, Fragility fractures, Clinical diagnosis of Paget’s disease of bone, Radiological diagnosis of Paget’s disease of bone, Biochemical diagnosis of Paget’s disease of bone, Genetics of Paget’s disease of bone, Complications of Paget’s disease of bone, Therapy of Paget’s disease of bone, Paget’s guidelines

## Abstract

**Introduction:**

Paget’s disease of bone is a focal skeletal disorder causing bone deformities and impairing bone quality. Despite the prevalence of asymptomatic cases is increasing, the progression of the disease can lead to invalidating complications that compromise the quality of life. Doubts on clinical and therapeutic management aspects exist, although beneficial effects of antiresorptive drugs, particularly bisphosphonates are known. However, limited information is available from randomized controlled trials on the prevention of disease complications so that somewhat contrasting positions about treatment indications between expert panels from the main scientific societies of metabolic bone diseases exist. This task force, composed by expert representatives appointed by the Italian Society of Osteoporosis, Mineral Metabolism and Skeletal Diseases and members of the Italian Association of Paget’s disease of bone, felt the necessity for more specific and up to date indications for an early diagnosis and clinical management.

**Methods:**

Through selected key questions, we propose evidence-based recommendations for the diagnosis and treatment of the disease. In the lack of good evidence to support clear recommendations, available information from the literature together with expert opinion of the panel was used to provide suggestions for the clinical practice.

**Results and conclusion:**

Description of the evidence quality and support of the strength of the statements was provided on each of the selected key questions. The diagnosis of PDB should be mainly based on symptoms and the typical biochemical and radiological features. While treatment is mandatory to all the symptomatic cases at diagnosis, less evidence is available on treatment indications in asymptomatic as well as in previously treated patients in the presence of biochemical recurrence. However, given the safety and long-term efficacy of potent intravenous bisphosphonates such as zoledronate, a suggestion to treat most if not all cases at the time of diagnosis was released.

## Background

Paget’s disease of bone (PDB, also called “osteitis deformans” or “Paget osteodystrophy”) is a focal skeletal disease that typically affects adults, causing pathognomonic deformities in one (monostotic form) or more (polyostotic form) skeletal sites [1, 2]. The skeletal sites most frequently affected by the disease are the pelvis (up to 70% of cases), the femur (30–55%), the lumbar spine (25–50%), the skull (20–40%) and the tibia (15–30%) [2]. In polyostotic forms, the distribution of lesions is typically asymmetrical. The appearance of new affected sites some years after the initial diagnosis is rare [3, 4]. PDB is considered a disease of osteoclasts that appear enlarged, hypernucleated and hyperresponsive to different stimuli such as 1,25 dihydroxyvitamin D, interleukin 6 and receptor activator of nuclear factor kappa-Β ligand (RANK-L) [5]. In addition, the functional interaction between osteoclasts and osteoblasts in the bone-remodeling units appears abnormal, leading to increased but disorganized bone formation. The altered bone turnover and overgrowth of pagetic bone often determines bone deformities, osteoarthritis (mainly affecting the joints adjacent to the pagetic lesion), fractures and pain. Other complications ascribed to PDB include neurological compression syndromes (particularly hearing loss in case of skull involvement), nephrolithiasis, high-output heart failure, vascular calcifications, and, in less than 1% of cases, neoplastic degeneration in osteosarcoma or, less frequently, giant cell tumor (GCT) (Table 1). Remarkably, over half of the GCTs arising on pagetic bone have been described in patients originating from Southern Italy, and particularly from Campania [6].

**Table 1:** Complications associated with Paget’s disease of bone

System	Complications		
	Common (10% or above)	Less common	Rare (< 1%)
Osteoarticular	Bone pain		
	Increase in bone size		Spinal cord stenosis
	Bone deformity		
	Osteoarthritis at adjacent joints		
	Pseudo-fractures and fractures		
Neurological	Headache	Hearing loss	Cranial nerve deficits
		Tinnitus	Basilar invagination
			Obstructive hydrocephalus
			Paraplegia, paraparesis
			Dementia (vascular steal syndrome)
Metabolic	Hyperparathyroidism*	Nephrolithiasis	Hypercalcemia
			Hyperuricemia
Cardiovascular		Endocardial calcifications	High output heart failure
		Aortic stenosis	
Neoplastic			Sarcomas
			Giant cell tumor

PDB is rarely diagnosed in subjects under the age of 40 and affects both sexes with a slight prevalence of males [1–3]. Its prevalence also increases with aging, so that it was estimated that up to 5–8% of subjects after the eighth decade of life may be affected by PDB in the geographical areas with the highest incidence. The disease is more common in Caucasians of European ancestry, but it has also been described in subjects of African ancestry, while it has been less frequently reported in Asiatic individuals [1, 7, 8]. The highest prevalence has been described in Great Britain, particularly in Lancashire [7–9], and in countries with high rates of immigration from Great Britain (Australia, New Zealand and the northeastern United States) [8]. In Italy, the prevalence of PDB is around 1% or below, with an area of high prevalence and greater severity in the rural regions of Campania [10, 11]. However, the most recent epidemiological studies have also demonstrated a progressive reduction of both the clinical severity and the onset of the disease compared to the past, with a higher frequency of monostotic cases [8, 12]. This may explain the marked decrease in the prevalence of new PDB diagnoses in the recent years [12].

A familial predisposition has been found in a variable number of patients with PDB, reaching 40% of cases in an in-depth survey from Spain [13], and mutations in different genes have been associated with the disorder [14]. Most of the associated genes, such as sequestosome 1* (SQSTM1),* tumor necrosis factor receptor superfamily member 11a *(TNFRSF11A),* valosin containing protein *(VCP),* and profilin 1 (*PFN1*)*,* are involved in the regulation of osteoclast formation and activity and thus might explain the peculiar characteristics of these cells in PDB [14]. This reinforce the hypothesis that the primary cellular abnormality of PDB resides in the osteoclast, and, indeed, antiresorptive agents are the treatment of choice for this disorder, since they suppress osteoclast activity and restore bone-remodeling rates toward normal. Despite the well documented effects of antiresorptives, and particularly the most potent bisphosphonates (BPs), on the improvement of pain and the suppression of the excessive bone turnover associated with PDB [15], there is limited information from randomized controlled trials (RCTs) about the effect of treatment on the prevention of pagetic complications (e.g., osteoarthritis, hearing loss or other neurological sequelae, deformity and neoplastic degeneration), although this sounds a reasonable supposition. This has led to somewhat contrasting positions about treatment indications that were given by expert panels from the Endocrine Society or the International Osteoporosis Foundation together with the European Calcified Tissue Society [16–18]. Importantly, PDB still remains an underdiagnosed and overlooked clinical condition [19].

## Purpose and scope

Based on the evidence provided above and despite the limited information from RCTs or observational studies, the members of this task force felt the necessity of providing more specific and up to date indications for the diagnosis and treatment of PDB. Thus, this position statement focuses on the optimal approach for an early diagnosis of PDB and its clinical management in different patient settings, in order to improve symptoms and to manage, if not prevent, the skeletal and extraskeletal complications.

Specifically, the following key questions have been addressed:

A. Diagnosis of PDB:

1. What diagnostic tests are necessary in a patient with a clinical suspicion of PDB?
2. In a patient with radiographic signs suggestive of PDB, which other tests are necessary for the diagnostic setting?
3. In a patient with high bone turnover markers (with or without specific symptoms) which diagnostic tests are appropriate to confirm or exclude the diagnosis of PDB?
4. In a patient with clinical suspicion of PDB, in case the diagnostic radiological criteria are not fully met, is a biopsy examination necessary?
5. In a subject with family history for PDB, is diagnostic screening indicated or not?
6. Is genetic testing recommended after PDB diagnosis?
7. In an adult subject with family members affected by PDB and carriers of known mutations (*SQSTM1*, *ZNF687* or *PFN1*) is mutational screening appropriate?

B. Treatment of PDB:

B.1 Who and When to Treat?

1. Is treatment needed in a newly diagnosed symptomatic PDB patient?
2. Is treatment needed in a newly diagnosed asymptomatic PDB patient?
3. Is biochemical follow-up necessary in a patient after a therapeutic cycle for PDB?
4. In a patient treated for PDB with persistent painful symptoms, is integration with analgesic therapy appropriate?
5. Is a new radiological examination indicated in a previously treated PDB patient, with exacerbation of painful symptoms in the site of a pagetic lesion?
6. Is antiresorptive retreatment indicated in a patient previously treated for PDB, with exacerbation of painful symptoms in the site of pagetic lesion?
7. Is antiresorptive retreatment indicated in a previously treated PDB patient with increased total alkaline phosphatase (or other marker of bone turnover)?
8. Is a therapeutic antiresorptive course indicated in a patient with PDB in anticipation of an orthopedic surgical procedure?
9. Is antiresorptive treatment necessary in an “immobilized” PDB patient?

B.2 How to Treat?

1. In a patient with newly diagnosed PDB requiring medical treatment, which antiresorptive agent should be preferred?
2. Is supplementation with calcium and/or vitamin D appropriate in a PDB patient on antiresorptive treatment?
3. Is it appropriate to change the therapeutic antiresorptive regimen in a previously treated PDB patient who is experiencing relapse of the disease (by clinical and/or biochemical point of view)?

All the above questions were specifically formulated in order to provide clear and up to date indications (based on clinical evidence or, failing that, on clinical expertise) about the diagnosis and management of PDB. This position statement is targeted towards all health professionals involved in the clinical management of patients with PDB, including endocrinologists, rheumatologists, orthopedics, internal medicine specialists, and general practitioners. The task force will conduct regular reviews every two years after publication of the position paper, to determine whether the evidence has progressed significantly enough both to alter the current recommendations and to require an update.

## Methodology

A national task force was composed by expert representatives appointed by the Italian Society of Osteoporosis, Mineral Metabolism and Skeletal Diseases (SIOMMMS) and members of the Italian Association of Paget’s disease of bone (AIP, https://www.pagetitalia.com). Position statement development included the following steps: (1) definition of the clinical questions; (2) search for literature sources; (3) evaluation of the clinical content of sources; (4) evaluation of the quality and coherence of sources; (5) setting-up of the recommendations; (6) external review of the position document and (7) adoption, endorsement and implementation of the position document.

The members of the working group were tasked to develop questions to be answered and to identify, consider and cite relevant evidence from existing systematic reviews and relevant publications, supplemented by the multi-disciplinary expertise of the appointed taskforce. To this regard, a systematic search of medical databases (PubMed, Cochrane Register and EMBASE) was performed until January 2022.

We used the Grading of Recommendations, Assessment, Development, and Evaluation (GRADE) system to describe the evidence quality and support the strength of the position statements provided by this taskforce on each of the selected key questions [20, 21]. Briefly, according to the GRADE system the evidence quality was categorized as high (+ + + +), moderate (+ + +), low (+ +), or very low (+). High-quality evidence was defined as consistent evidence from well-performed RCTs or exceptionally strong evidence from unbiased observational studies. Moderate-quality evidence was evidence from RCTs with important limitations (inconsistent results, methodological flaws, or indirect or imprecise evidence) or unusually strong evidence from unbiased observational studies. Low-quality evidence was evidence for at least one critical outcome from observational studies, RCTs with serious flaws, or indirect evidence. Very low-quality evidence was evidence for at least one of the critical outcomes from unsystematic clinical observations or very indirect evidence. The GRADE system classifies the strength of recommendations into two grades (strong or weak). Strong recommendations (terminology: “we recommend”) mean that benefits clearly outweigh harms and burdens. Weak recommendations (terminology: “we suggest”) mean that the desirable effects of adherence to a recommendation probably outweigh the undesirable effects, but the panel is not confident. If the panel believes that benefits and harms are closely balanced, or significant uncertainty exists about this balance, a weak recommendation is released. Basically, high-level evidence supports strong recommendations, whereas biased or low-quality evidence generate weak recommendations. However, making recommendations requires considering other factors, such as patients’ values and preferences, local circumstances, and clinical expertise. Integrating quality of evidence and other considerations is necessary when a clinical recommendation is released for use in practice. As consequence, the strength of a recommendation can be downgraded (weak recommendation generated by high- or moderate-quality evidence) or upgraded (strong recommendation generated from low- or very-low-quality evidence). The GRADE system formally recognizes this possibility.

All the authors contributed to the writing of the manuscript and the final draft statement was agreed to by all the authors. The draft statement was then submitted to representative members of the Councils of the SIOMMMS, who provided feedback and gave the final approval.

## Results and recommendations

### Diagnosis of PDB

Given the above-mentioned secular trends, with a reduced prevalence and severity of PDB, most PDB cases are now asymptomatic or pauci-symptomatic and thus diagnosis is often made casually following investigations made for other clinical reasons [1, 2]. The most common clinical presentation is bone pain (which is often persistent), followed by bone deformity, or other symptoms such as deafness or pathological fractures. Albeit the occurrence of these symptoms is variable among the different reports in the literature, prevalence estimates were described by a large systematic review involving 4215 patients at PDB diagnosis, concerning bone pain (38.0%), bone deformity (20.3%), fractures (10.6%), and deafness (5.9%) [22]. An additional clinical feature of PDB is related to the increased blow flow of affected pagetic sites, so that the overlying skin often appears warm to the touch.

**Fig. 1:**
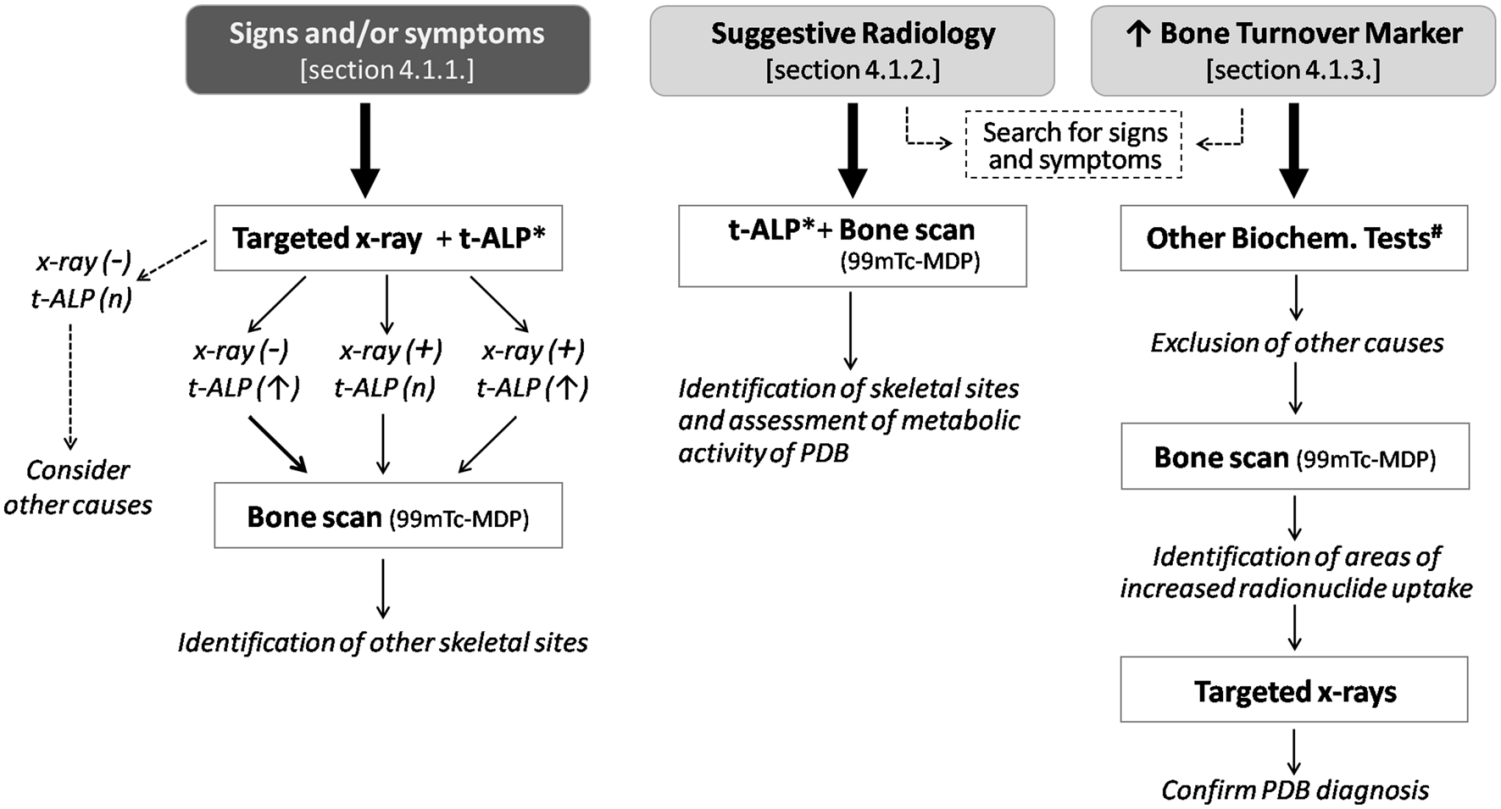
Diagnostic flowchart of Paget’s disease of bone under different clinical settings. The diagnostic process changes in relation to the presence or absence of signs and/or symptoms of disease. In asymptomatic disease, the clinical hypothesis is dependent on the presence of suggestive radiological features (from X-ray, CT, or MR analyses performed for other purposes) or the incidental finding of increased total alkaline phosphatase (t-ALP) or any other marker of bone turnover. In the setting of inconclusive radiological and biochemical findings, a bone biopsy may be indicated to confirm diagnosis. *T-ALP or, alternatively other markers of bone turnover (e.g., B-ALP and P1NP); # first level biochemical tests (plasma and urinary calcium and phosphate, renal function indices, protein electrophoresis, liver function tests) and, eventually, parathyroid hormone and 25OH vitamin D

In the absence of these signs and/or symptoms the suspected diagnosis of PDB generally derives from suggestive skeletal features at radiological tests (generally X-rays, but also CT, or MRI, often performed for other diagnostic purposes) or due to the incidental finding of elevated levels of total alkaline phosphatase (t-ALP) in the presence of normal liver tests. A graphical flowchart summarizing the recommendations for PDB diagnosis under the different clinical settings is given in Fig. 1.

#### What diagnostic tests are necessary in a patient with clinical suspicion of PDB?

The clinical suspicion of PDB generally arises from the presence of localized bone pain, especially if associated with the finding of bone deformities in one or more skeletal sites or other symptoms such as deafness. Under these circumstances, confirmatory diagnosis is essentially based on targeted radiological exams of the suspected skeletal site(s) and the detection of an increase in the markers of bone turnover [2]. To date, there are few studies that specifically examined and compared the diagnostic accuracy of radiological and biochemical tests and none of them established a priority chronological order in their execution. One of these studies was performed in the population-based setting of pagetic and non-pagetic cases from the Rotterdam Study cohort, showing that, albeit t-ALP was an excellent marker of the disease (equivalent to a relative risk for PDB of 10.9 in the presence of raised serum levels), systemic radiographs (including thoracic and lumbar spine, pelvis, proximal femurs, knees, wrists, and hands) were much more sensitive for PDB diagnosis [23]. In fact, a relevant number of PDB cases of that cohort (42%) had normal t-ALP.

**Fig. 2:**
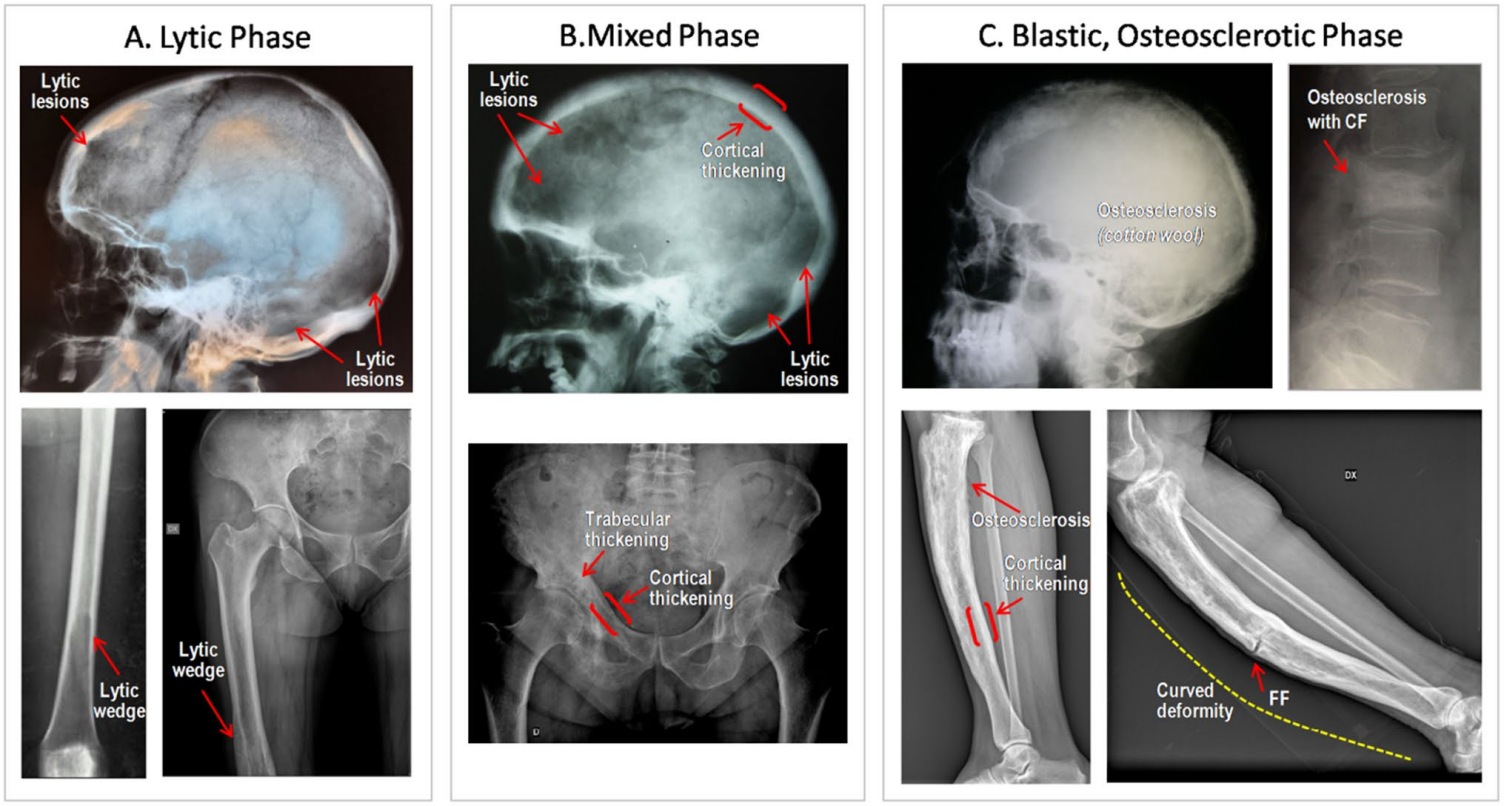
Radiological presentation of Paget’s disease of Bone. **A** Lytic phase. Upper panel: circumscribed osteolytic skull lesions in the frontal and occipital regions. Lower panels: osteolytic lesions of the distal femur progressing proximally and assuming the shape of a flame or inverted V. **B** Mixed phase. Upper panel: circumscribed osteolytic skull lesions, associated with marked thickening of the diploic space; lower panel: extensive involvement in the right hemipelvis with areas of cortical (ilio-pectineal and ilio-ischial lines) and trabecular thickening and circumscribed osteolytic lesions. **C** Blastic, osteosclerotic phase. Upper panel: marked thickening of the cranial table, particularly the inner calvarial table, together with several areas of focal sclerosis (“cotton wool” appearance) [right]; compression fracture (CF) at a sclerotic pagetic vertebra [left]. Lower panels: pagetic tibias in blastic phase, with diffuse cortical thickening, trabecular osteosclerosis (causing a loss of distinction between cortex and medulla), bone enlargement and deformity. In the right panel is shown a transverse fissure fracture (FF)

Radiographically, PDB may be characterized by areas of osteolysis (with advancing resorption wedge), thickening of cortical bone, accentuation and coarsening of trabecular pattern along stress lines, loss of distinction between cortical bone and marrow, osteosclerosis, enlargement of bone contours and bone deformity (Fig. 2). None of the above radiographic findings considered individually is, however, pathognomonic of the disease, but their combination is often diagnostic, as is the asymmetric distribution of skeletal lesions in patients with polyostotic PDB [24, 25].

Moreover, X-rays’ analysis may give some indications about the progress of the disease, with the characterization of one of the three typical phases of PDB: (a) *lytic phase *(Fig. 2A), the early phase of PDB characterized by large, well-defined areas of osteolysis, but in absence of peripheral sclerosis, a feature that is common to others pathologies (e.g., fibrous dysplasia or metastatic disease), therefore, requiring others tests, as computed tomography (CT) and bone biopsy, to confirm the diagnosis [26]; (b) *mixed phase *(Fig. 2B), the most frequently observed presentation of PDB, with evidence of most cardinal radiological features and, therefore, strongly diagnostic; and (c) *blastic, osteosclerotic phase *(Fig. 2C), corresponding to the so-called burnt phase at bone scintigraphy, with extensive areas of osteosclerosis causing a loss of distinction between cortex and medulla, bone enlargement and deformity. The duration of each phase is variable and hard to define, since they are part of a continuous spectrum and may coexist in one bone at the same time.

Standard radiographs are widely available and inexpensive examinations, allowing the identification of stress fractures which most typically occur in deformed pagetic bones subjected to mechanical loading. They are, therefore, to be considered first level tests in patients with clinical suspicion of PDB.

Once supported by radiological evidence, the diagnostic procedure must include the assessment of bone turnover (generally, t-ALP) and the identification of other skeletal sites possibly affected by PDB (see question “Sect "In a patient with radiographic signs suggestive of PDB, which other tests are necessary for the diagnostic setting?. below for further details on the use of bone turnover markers” for more details). The latter is generally achieved through a whole-skeleton bone scan for mapping the metabolically active areas of the disease.

**Table:** 

Quality of evidence (GRADE)	Clinical recommendation	Strength of recommendation
GRADE + +	In the clinical suspicion of PDB, it is recommended to perform a radiographic examination of the site that led to the clinical suspicion, together with a dosage of total alkaline phosphatase (or alternatively another bone-remodeling marker)*	Strong (positive)

see Sect "In a patient with radiographic signs suggestive of PDB, which other tests are necessary for the diagnostic setting?. below for further details on the use of bone turnover markers"

#### In a patient with radiographic signs suggestive of PDB, which other tests are necessary for the diagnostic setting?

In patients with radiographic signs suggestive of PDB (often as a casual finding in the context of a diagnostic screening for other clinical conditions), the whole-body bone scan with technetium-99 m labeled methylene diphosphonate (99mTc-MDP) represents the first level diagnostic tool for evaluate disease extension (namely, the number of skeletal sites involved by PDB) [27–29]. This is generally associated with an assessment of bone turnover status.

**Fig. 3:**
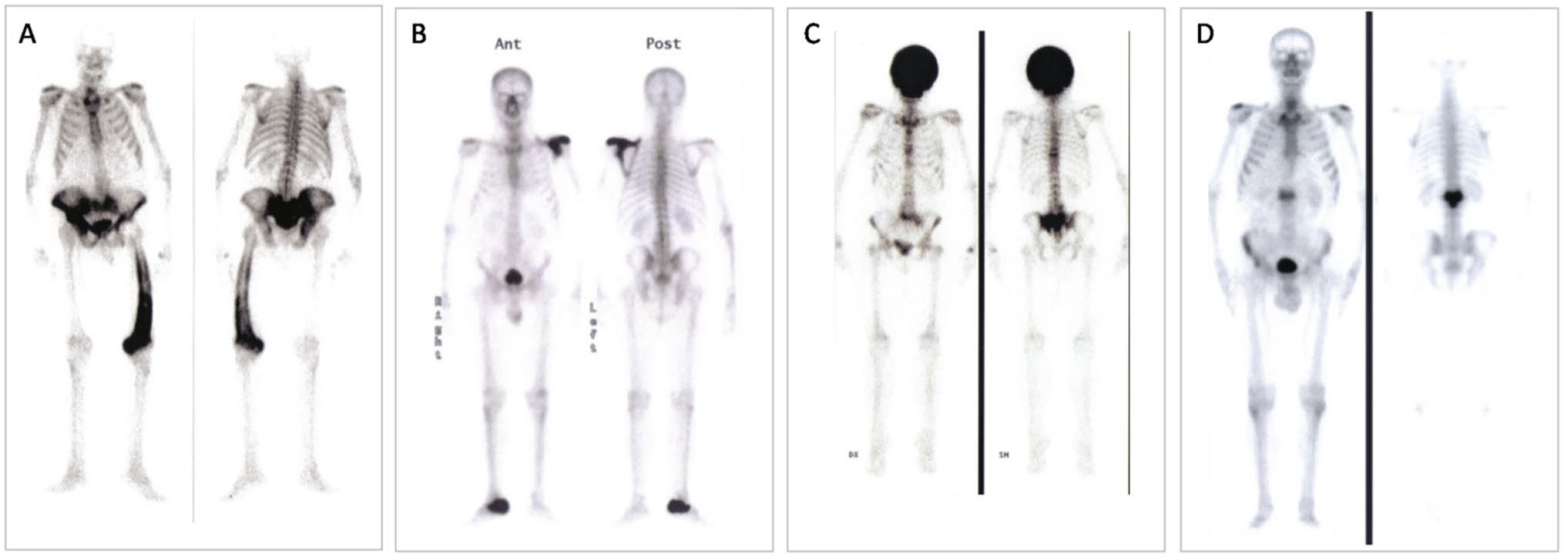
Bone scan features of Paget’s disease of bone. **A** Whole-body bone scan with technetium-99 m labeled methylene diphosphonate (99mTc-MDP) is required to identify areas of increased metabolic activity that are suggestive of pagetic sites. Some pathognomonic features may be the involvement and enlargement of a whole skeletal district (2B and 2C), the marked deformity, more easily detected in long bones (2A), and the so-called “Mickey mouse” shape of a vertebral body (2D)

The radiolabeled 99mTc-MDP binds to skeletal sites with increased metabolic activity, thus revealing the bones affected by metabolically active PDB [27]. When long bones are involved, the radiolabeled bisphosphonate binds first the epiphysis and after the diaphysis [29]. Importantly, bone scan identifies the sites involved by PDB even before they show any radiological and/or clinical sign [30]. Despite several clinical conditions may cause focal hyper-accumulation of 99mTc-MDP (e.g., arthritis, bone metastasis, infections, etc.), the scintigraphic features of PDB can be pathognomonic (e.g., “clover” and “heart sign” of the spine) [Fig. 3], even though false positive results have been also described [31, 32].

However, despite the better sensibility of whole-body bone scan compared to standard X-ray for the diagnosis of PDB, sclerotic (“burned out”) lesions may not bind the radiolabeled bisphosphonate. This occurs in 2–10% of bones with radiological signs of PDB [30–34]. Of interest, information derived from a study by Guañabens and colleagues also suggested that, in case the 99Tc bone scan is not available, standard X-rays of abdomen, skull, and both tibiae are effective for detecting pagetic lesions, increasing diagnostic sensitivity to 93%, as compared to 79% of a plain abdominal X-ray [35]. Recently, it has been suggested that 18F-sodium fluoride positron emission tomography (18F-NaF-PET) may be of great potential in detecting and monitoring PDB, even in asymptomatic form, since it is sensitive to the increased osteoblastic activity observed in pagetic bone [36]. In fact, the dissociated 18F- can be incorporated into hydroxyapatite crystals and its uptake may reflect both the osteoblastic activity and bone perfusion, allowing for the quantification of bone turnover. However, albeit this technique might potentially offer some diagnostic benefits over 99Tc bone scan, more specific clinical controlled studies will be needed to confirm/validate this method before its use can be recommended in the diagnostic setting of peculiar cases of PDB.

Concerning biochemical markers of bone turnover, Al Nofal and colleagues performed a systematic review and metanalysis of 17 observational studies and 1 trial, overall involving 953 patients with previously untreated PDB, to evaluate the relationship between disease activity, assessed by bone scan, and the levels of the following bone turnover markers: t-ALP, bone-specific alkaline phosphatases (B-ALP), procollagen 1 Intact N-Terminal Propeptide (P1NP), serum carboxy-terminal peptide of type 1 collagen (sCTx), urinary carboxy-terminal peptide of type 1 collagen (uCTx), and urinary amino-terminal peptide of type 1 collagen (uNTx)] [37]. A significant and direct relationship between the levels of all bone turnover markers and disease activity was demonstrated, with correlation indices ranging between 0.58 and 0.80, without major differences between each of the markers. The best correlation indices were reported for P1NP, uNTx and b-ALP. However, considering the higher cost and low availability of these markers, we generally recommend the use of serum t-ALP levels together with common indices of liver function as first level biochemical marker for assessing PDB activity. When biochemical markers of liver function are elevated, and/or when t-ALP levels are within the normal range in the clinical or radiological suspicion of PDB, we thus recommended the measurement of more sensitive markers such as P1NP or b-ALP [38]. This diagnostic procedure is particularly recommended in patients with monostotic disease involving small bones.

**Table:** 

Quality of evidence (GRADE)	Clinical recommendation	Strength of recommendation
GRADE: + +	In patients with radiographic signs of PDB, we recommended to perform a whole-body bone scan (99mTc-MDP), and the measurement of bone turnover markers to evaluate the extension and the metabolic activity of the disease	Strong (positive)

#### In a patient with high bone turnover markers (with or without specific symptoms), which diagnostic tests are appropriate to confirm or exclude the diagnosis of PDB?

Considering studies assessing the sensitivity of plain radiography, bone scintigraphy and bone turnover markers in diagnosing PDB, a combination of these methods guarantees the highest diagnostic accuracy [33–35, 37, 38]. Thus, when PDB is suspected in a patient with an isolated elevation of t-ALP or other bone turnover markers, a radionuclide bone scan imaging (99mTc-MDP) is first recommended, as this is more sensitive than plain X-rays in the identification of pagetic lesions [30, 34]. Then, targeted X-rays of the areas of increased radionuclide uptake are also recommended, in order to identify the radiological features of PDB (as outlined in paragraph “What diagnostic tests are necessary in a patient with clinical suspicion of PDB?”). If bone scans are not readily available, we suggest plain X-rays of the abdomen (including the lower ribs and femoral heads), both tibias, the skull, and facial bones since X-rays of these sites have been found to detect PDB in 93% of patients [35]. Indeed, radionuclide bone scans are more expensive and have an effective radiation dose up to 3–5.36 mSv, which is much higher than radiation dose of X-rays (1.09 mSv).

It should be, however, emphasized that the isolate finding of an increase in one or more markers of bone turnover does not always indicate a suspected diagnosis of PDB and, therefore, in the diagnostic process, in order to exclude other skeletal conditions of high bone turnover (e.g., hyperparathyroidism, fibrous dysplasia, multiple myeloma, skeletal metastases and primitive skeletal neoplasms), it is often necessary to perform first level biochemical tests (plasma and urinary calcium and phosphate, renal function indices, protein electrophoresis, liver function tests) and subsequently any second level tests. In particular, the measurement of parathyroid hormone (PTH) and 25OH vitamin D (25OHD) levels can be of relevance for diagnostic and therapeutic purposes. These biochemical tests should be preferentially performed before proceeding with the radiological and bone scan analyses, particularly in the absence of signs and symptoms suggestive of PDB.

To date, few studies have been performed to assess the role of other imaging techniques, such as magnetic resonance imaging (MRI) and computed tomography (CT), in diagnosing PDB.

Roberts et al. compared data collected from MRI, CT, and plain radiographs of 13 patients with PDB, reporting highly consistent data across the 3 imaging methods [39]. Although the use of MRI and CT does not generally appear necessary for the diagnosis of PDB, the information provided by these imaging methods can be very useful in some clinical settings, for an evaluation of various disease complications and particularly in case of neurological manifestations (nerve compression, hydrocephalus) or to exclude malignancies [40–42].

**Table:** 

Quality of evidence (GRADE)	Clinical recommendation	Strength of recommendation
GRADE: + +	In the patient with an increase in one or more markers of bone turnover (particularly in the absence of specific signs or symptoms), it is first recommended to screen routine blood chemistry tests (plasma and urinary Ca and P, protein electrophoresis, renal function, liver function tests) and assess PTH and 25OHD levels. In case these analyses lead to an increased diagnostic suspicion, then proceed with the bone scan and/or radiological screening extended to the most frequently affected sites (skull, spine, pelvis and tibia, when bone scan is not performed)	Strong (positive)

#### In a patient with clinical suspicion of PDB, in case the diagnostic radiological criteria are not fully met, is a biopsy examination necessary?

In some cases, both the radiological examination and the other diagnostic tests for PDB (described above) remain inconclusive and are unable to exclude other pathologies (Table 2). In this setting, a biopsy may be indicated to confirm the diagnosis, even though the use of MRI or CT, due to their additive role in the exclusion of other disorders, can be indicated before proceeding with the biopsy. In example, radiological lesions of the skull of patients with fibrous dysplasia may often be confused with PDB. In a specific study, Tehranzadeh et al. compared skull CT images obtained in 10 PDB cases and 10 patients with fibrous dysplasia, identifying some features suggestive of PDB (e.g., symmetrical involvement of the cranial bones and of thickness of the cranial cortices) and others suggestive of fibrous dysplasia (ground glass appearance of the skull bones and involvement of the sinuses, sphenoid, orbit, and nasal passages) [43]. Considering that PDB patients are at an increased risk of developing primary bone neoplasms compared to non-pagetic subjects [6, 44, 45], a bone biopsy procedure can be also recommended when X-rays, MRI and/or CT lead to the suspicion of neoplastic degeneration.

**Table 2:** Main bone disorders to be excluded in the diagnostic process for Paget’s disease

Disease	Radiological features differing from PDB
Bone metastasis	Ill-defined osteoblastic or lytic lesions without cortical thickening and bone enlargement
Chronic, non-bacterial osteomyelitis	Inhomogeneous osteosclerosis and/or sequestrum formation (necrotic bone)
Fibrous dysplasia	Homogeneously sclerotic lesion with “ground glass like appearance”. No bone expansion or cortical breach
Hyperostosis frontalis interna	Usually affects the outer calvarial table more prominently
Erdheim Chester disease	Osteosclerotic lesions are generally symmetrical and do not lead to bone deformity

The largest description of histomorphometry and histological characteristics of pagetic bone has been provided by Seitz and colleagues that examined bone biopsies from 754 patients [46]. Histologically, PDB is characterized by an increase in cancellous bone volume secondary to an increase in trabecular number, rather than an increase in trabecular thickness, a sixfold increase in osteoid volume, massive fibrosis at the trabecular bone surface, and an increase in the number and volume of osteoclasts. In addition to conventional biopsy, fine-needle aspiration biopsy is an accurate, safe, efficient, well-tolerated, and affordable method for diagnosing primary bone tumors, such as osteosarcoma [47].

**Table:** 

Quality of evidence (GRADE)	Clinical recommendation	Strength of recommendation
GRADE: +	When the radiological analyses (X-rays, CT and/or MRI) do not provide univocal and pathognomonic findings of the disease, a targeted biopsy examination is suggested (unless specifically contraindicated)	Weak (positive)

#### In a subject with family history for PDB, is diagnostic screening indicated?

Regardless of the recent identification of germline mutations in specific genes in sporadic and/or familial forms of PDB [14], there is ample evidence that from 12 to 40% of patients have at least one first-degree relative affected by the disease [13, 48, 49]. Moreover, estimates deriving from epidemiological surveys suggested a 7–10 times increased risk of developing the disease in first-degree relatives of a pagetic patient, compared to that calculated for the general population [48, 49]. This risk appears even greater in relatives of PDB cases with deforming disease and/or with a reported diagnosis at an early age [48, 50]. Therefore, from a practical point of view, even in the lack of specific comparative studies among the different diagnostic tools, it can reasonably be suggested that all first-degree relatives of pagetic patients should in any case undergo a periodic screening of at least t-ALP (or other bone turnover markers), generally starting from 40 years of age, or earlier if the affected relative had an onset of the disease at an even earlier age. This will make possible to carry out adequate surveillance and to identify early the presence of PDB (especially polyostotic forms), hopefully still in a pre-/asymptomatic form, and thus establish any appropriate therapy in order to prevent disease progression [51]. In fact, the assessment of t-ALP appears as the simplest, largely available, cheapest, appropriate, and thus cost-effective tool as compared to the measurement of other bone turnover markers, or the use of X-rays and bone scan. In case high t-ALP levels are identified (or in any case of increased bone turnover), the diagnostic procedure to be followed will be the same described above, in section 4.2.3. Concerning the use of t-ALP for this specific setting, it should be, however, underlined that, albeit it represents a valuable marker, normal t-ALP levels can be also found in patients with PDB, particularly in case of monostotic forms [23], as well as in asymptomatic cases with early PDB and a positive family history [52]. In this respect, either uNTx or P1NP demonstrated a better predictive value in identifying early cases with pagetic lesions [52]. In case of negative results, we suggest that the screening of ALP or, eventually, other bone turnover markers can be repeated periodically, approximately every 3 years.

**Table:** 

Quality of evidence (GRADE)	Clinical recommendation	Strength of recommendation
GRADE: +	In a subject with documented family history of PDB, it is suggested to monitor the levels of t-ALP, or other markers of bone turnover, even in the absence of specific symptoms	Weak (positive)

#### Is genetic testing recommended after PDB diagnosis?

Germline mutations in *SQSTM1* are reported in up to 50% of familial cases of PDB (as well as in up to 10% of sporadic cases) and have been often related to an increased disease severity and an earlier onset [53–55]. Although rarely observed, also *PFN1* and *ZNF687* mutations are generally associated with severe, polyostotic forms of the disease, with a disease onset between the third and fourth decade of age [14]. Moreover, *ZNF687* mutations have been associated to a particularly increased risk of neoplastic degeneration in GCT, while either GCTs or osteosarcomas have been often described in the pedigrees with *PFN1* mutation [14, 56, 57]. To date, specific guidelines addressing when and why perform genetic screening in PDB are still lacking and certainly additional information is required to address whether genetic testing may have a good cost–benefit ratio for all PDB patients [51]. However, based on the available clinical information, we suggest that genetic screening could be performed in familial PDB cases and in all PDB patients with early onset (< 50 years), polyostotic PDB. The latter indication is further strengthened by the higher risk of neoplastic degeneration in the presence of early-onset PDB related to *ZNF687* or *PFN1* mutation.

**Table:** 

Quality of evidence (GRADE)	Clinical recommendation	Strength of recommendation
GRADE: +	We suggest that genetic screening can be carried out in familial PDB cases and in all PDB patients with early onset (< 50 yrs), polyostotic PDB	Weak (positive)

#### In an adult subject with family members affected by PDB and carriers of known mutations (SQSTM1, ZNF687 or PFN1), is mutational screening appropriate?

Although it was originally reported that up to 90% or more of unaffected *SQSTM1* mutation carriers from pagetic families develop the disorder by age 65 years [58], lower penetrance rates have been described in different settings, suggesting that only a proportion of *SQSTM1* mutation carriers will develop PDB, and possibly at a later age than their affected relatives [59–67]. Indeed, in a recent screening of baseline clinical, radiological and bone scan characteristics of 222 apparently unaffected carriers of *SQSTM1* mutations (from known PDB families) who took part in the Zoledronic acid in the Prevention of Paget’s disease (ZiPP) study, asymptomatic PDB was confirmed in about 9% of cases [52]. Importantly, in the very recent report of that trial, after a median duration of 84 months, zoledronate-treated subjects did not develop any new pagetic lesion compared to placebo [68]. An improvement of existing lesions (completely disappearing on bone scan in 87% of cases) was also demonstrated in zoledronate-treated subjects versus placebo. This suggests that genetic testing of *SQSTM1* mutation coupled with prophylactic zoledronate treatment has a favorable effect on the development and progression of PDB.

Thus, while waiting for more detailed information, we suggest mutational screening in all first-degree relatives of PDB patients with known *SQSTM1* mutations. Then, in case a mutation is identified, the genetic screening should be extended to the other relatives, in order to identify additional asymptomatic carriers and possibly at an early age, before they express, clinically and/or biochemically, the disease itself. In fact, unlike clinical tests, genetic testing does not require serial repetitions.

Moreover, a genetic screening of family members of PDB cases with either *PFN1* or *ZNF687* mutation is particularly advised, if not mandatory, given the related risk of neoplastic degeneration. Indeed, in a recent analysis of a large pedigree, with severe, early onset, PDB associated with *PFN1* mutation, the extension of the genetic screening to young, fourth generation, relatives allowed the identification of a 17-year-old female mutation carrier, who was then affected by PDB (despite the young age), with initial osteolytic lesions at the skull and the right tibia [57]. She then underwent intravenous bisphosphonate treatment in order to hopefully arrest the progression of disease.

**Table:** 

Quality of evidence (GRADE)	Clinical recommendation	Strength of recommendation
GRADE: +	In a subject with one or more family members affected by PDB and carriers of *SQSTM1*, *ZNF687*, or *PFN1* mutations, where available, mutational screening is suggested	Weak (positive)

### Treatment of PDB

Pharmacological therapy of PDB involves the use of bone resorption inhibitors (usually BPs) combined or not with drugs for the management of painful symptoms (usually analgesics and non-steroidal anti-inflammatory drugs [NSAIDs]) [2, 15]. Nitrogen-containing BPs (N-BPs) are currently the treatment of choice of PDB; they are a versatile group of compounds that can be administered orally, intravenously, or as intramuscular injection [15, 69, 70]. An updated list with the commonly used N-BPs regimens for the treatment of PDB is given in Table 3.

**Table 3:** Commonly used N-BPs regimens for the treatment of Paget’s disease

Bisphosphonate	Administration route	Suggested dose and duration	Approval for PDB
Alendronate	Oral	40 mg/day for 2–6 months	FDA
Risedronate	Oral	30 mg/day for 2 months 17.5 mg/day for 8 months	FDA, EMA, AIFA, MHRA PMDA
Pamidronate	Intravenous	30–60 mg/day intravenously for 3 consecutive days (multiple treatment courses can be required)	FDA
Neridronate	Intravenous Intramuscular	100 mg for 2 consecutive days 25 mg/weekly for 2 months	AIFA AIFA
Zoledronate	Intravenous	5 mg by single infusion	FDA, EMA, MHRA, AIFA

*FDA* Food and drug administration (USA), *EMA* European medicines agency, *AIFA* Italian medicines agency, *MHRA* Medicines and healthcare products regulatory agency (UK), *PMDA* Pharmaceuticals and Medical Devices Agency (Japan)

There are currently conflicting positions regarding which patients to treat and when to treat. While previous expert reports and the clinical practice guidelines from the Endocrine Society suggest treating all patients with active disease (as established from an increase in one or more biochemical markers of bone remodeling), in the presence of specific symptoms (i.e., bone pain) or in asymptomatic cases with a greater risk of complications (e.g., involvement of weight bearing bones or immobilized patients) [16], more stringent criteria were given by an expert panel from the International Osteoporosis Foundation together with the European Calcified Tissue Society [17]. The latter, in the absence of sufficient evidence to support all the Endocrine Society indications, mainly suggested bone pain as the leading therapeutic indication, thus promoting a treatment strategy aimed at improving symptoms over a treat-to-target strategy aimed at normalizing bone turnover. To provide more clear indications, this taskforce separately addressed the main clinical settings on the basis of which to decide whether to treat and how to treat PDB.

#### Who and when to treat?

##### Is treatment needed in a newly diagnosed symptomatic PDB patient?

Most if not all guidelines and position papers about the clinical management of PDB are in general agreement that a PDB patient with a newly diagnosed, metabolically active disease and bone pain should be considered for N-BP therapy [1, 2, 16, 17]. In fact, the effectiveness of N-BPs in reducing the levels of t-ALP and/or other bone-remodeling markers to normal is well established [2, 15, 69–73]. In addition, the recent development of potent compounds administered intravenously as a single cycle (such as zoledronate or neridronate given in, respectively, 1 or 2 consecutive days) allows a long-term biochemical remission of PDB, with treatment-free intervals generally exceeding 5 years in most patients treated with zoledronate [73–77]. Indeed, in a small observational analysis of 107 elderly patients treated with a single intravenous infusion of zoledronate, only 14% had biochemical relapse 9 years after treatment (as established by an increase in P1NP levels above the normal range) [78]. At the same time, numerous evidences from randomized and non-randomized clinical trials demonstrated the efficacy of treatment with BPs, and particularly N-BPs, on the control of pain symptoms at the level of pagetic lesions [15].

A recent Cochrane revision and meta-analysis of the main “placebo-controlled” clinical trials relating to the use of BP in PDB further demonstrated the efficacy of this class of drugs in reducing bone pain, with an efficacy demonstrated in 45% of cases compared to the 23% observed in the placebo group (RR 1.97, 95% CI 1.29–3.01; NNT 5, 95% CI 2–15) [72]. However, most of the studies included in the meta-analysis referred to therapeutic cycles with first- and second-generation BPs such as etidronate and tiludronate, which are rarely used in clinical settings today, so it is conceivable that the more recent and powerful N-BPs may have a greater efficacy in the control of pain symptoms, as suggested by some comparative studies. In particular, the results of two recent randomized studies showed that a single intravenous infusion with zoledronate or neridronate (in one of these studies) was superior to therapeutic cycles with, respectively, risedronate (30 mg/ day orally for 2 months) [73–75] or pamidronate (30 mg intravenously for 2 consecutive days, every 3 months) [76] on the reduction of pain symptoms at 6 months and over the long term. On the other hand, there is limited evidence unequivocally confirming the efficacy of BP therapy on quality of life (except for the component linked to pain symptoms), on the prevention of deformity or other symptoms and complications associated with the disease. The only indications in this regard come from observational studies on limited series of patients which would suggest a certain efficacy of antiresorptive therapy on skeletal deformity of the cranial bones, hearing loss or neurological dysfunction associated with pagetic localization [79, 80].

**Table:** 

Quality of evidence (GRADE)	Clinical recommendation	Strength of recommendation
GRADE: + + +	Based on the available evidence, a course of N-BP therapy is recommended in all patients with newly diagnosed, symptomatic PDB, unless there are contraindications to treatment (e.g., clinically significant renal impairment) Both zoledronate and neridronate administered intravenously have shown greater efficacy in the control of pain symptoms	Strong (positive)

##### Is treatment needed in a newly diagnosed asymptomatic PDB patient?

As outlined above, there are some controversies about the necessity of antiresorptive treatment in all PDB patients with asymptomatic disease since evidence from randomized studies is very limited. These controversies have been somewhat fueled further with the results from a randomized clinical trial “The Paget’s Disease, Randomization Trial of Intensive versus Symptomatic Management Study” (PRISM), comparing the efficacy of “intensive” N-BPs regimens for biochemical disease remission versus to “symptomatic” therapy, carried out exclusively for the control of pain, in 1324 patients followed up prospectively for 3 years [81]. In fact, no significant differences in terms of quality of life, reduction of pain symptoms or possible complications (e.g., impaired hearing ability, the occurrence of fractures and the need for orthopedic surgery) were observed between the two treatment regimens. Similar results emerged in the extension study (PRISM-EZ), performed on 502 cases which extended the observation to approximately 7 years of follow-up, in which the use of zoledronate was privileged [82]. The authors of the study, therefore, underlined that the use of therapeutic N-BPs cycles for the sole purpose of guaranteeing biochemical remission of the disease, regardless of the presence or absence of symptoms, does not produce any benefit compared to symptomatic treatment, done exclusively in the presence of bone pain. In this regard, however, it must be emphasized that more than 70% of the cases were patients who had already undergone previous BP treatment (and thus not at PDB diagnosis) and with an advanced form of the disease along with its complications (e.g., hearing loss in 22%, fractures in 39%, bone deformity in 36%, and previous orthopedic surgery in 16%). Furthermore, for ethical reasons, a placebo group was obviously not included in the PRISM study, so it was not possible to assess the benefits of both therapeutic regimens compared to the absence of treatment.

Indeed, it is in any case well documented that, if left untreated, pagetic lesions generally undergo a progressive evolution (with an estimated progression of the lytic wedge of about 1–2 cm per year) [83], with a likely increase in the degree of bone deformity and the risk of complications such as fractures, osteoarthritis, or neurological syndromes [84]. This occurs particularly in the case of involvement of the skull, spine, pelvis, and the long bones. In contrast, antiresorptive treatment, together with a normalization of bone turnover markers, has been associated with a reduction in disease activity, as assessed by the reduction in isotope uptake at bone scan images [85, 86], or improvements in radiographic characteristics (e.g., with the filling of osteolytic areas and a decreased extension rate of the pagetic lesions) [87–91], as well as with the recovery of normal lamellar patterns of bone deposition on bone biopsy specimens [89, 92]. Moreover, in a small prospective 12-month observation in 41 PDB patients receiving BP treatment, an increased prevalence of pagetic complications was described in those patients whose bone turnover marker levels were lowered but not normalized [93].

Thus, albeit in the lack of definitive information from RCTs, based on those evidences and given the availability of safe and effective N-BPs (now allowing disease remission over the long term, if not life-long), this panel suggests that, in the absence of contraindications, most, if not all patients should undergo a N-BP treatment course at PDB diagnosis.

**Table:** 

Quality of evidence (GRADE)	Recommendation	Strength of recommendation
GRADE: +	Although there is no information from RCTs, given the long-term efficacy and safety of current therapeutic regimens, and given disease progression in untreated patients, a treatment course with N-BPs is suggested in all asymptomatic patients with newly diagnosed PDB, unless there are contraindications to treatment	Weak (positive)

##### Is biochemical follow-up necessary in a patient after a therapeutic cycle for PDB?

All RCTs on PDB considered as the primary endpoint the decrease in t-ALP and/or other markers of bone turnover. Thus, to assess the response to treatment and disease activity, we recommend to at least evaluate t-ALP levels or, as an alternative, other markers such as P1NP, uNTx and b-ALP [1–3]. Generally, the markers of bone resorption such as NTX and CTX show a more rapid decrease (between 10 and 20 days from N-BP treatment), while markers of bone formation (including t-ALP) have a slower decrease (2–3 months from N-BP treatment) [71, 73]. However, there is no clear evidence that any of the other markers are superior to t-ALP in PDB [71]. Indeed, osteoblast markers such as t-ALP, B-ALP and P1NP all show a performance approaching that of bone scintigraphy [71, 86]. Conversely, NTX, albeit considered as a reliable bone resorption marker in different conditions, appears less sensitive to the above-mentioned bone formation markers in detecting the effects of therapy in PDB [71]. Even though there is no general consensus on how to define the “therapeutic response” to antiresorptive treatment in PDB, most of the recent RCTs considered the normalization of serum ALP levels or a reduction of at least 75% in the ALP excess as an adequate indicator of response to N-BPs [2]. However, it has also been suggested that in order to maximize the duration of disease remission, t-ALP or any other chosen bone marker should be reduced below the midpoint of the reference range [2, 16]. In fact, a t-ALP reduction below the lower half of the reference range was associated with the maintenance of the therapeutic response for up to 6.5 years in more than 90% of patients treated with a single 5 mg zoledronate infusion [75].

Generally, a first follow-up of t-ALP could take place between 3 and 6 months after treatment, or as an alternative every 6–12 months, particularly in case of intravenous therapy with zoledronate or neridronate. In case of recurrence of disease activity during the follow-up (e.g., increased t-ALP levels), especially in the presence of painful symptoms, a decision about a possible retreatment is mandatory [2, 16] (see “Is antiresorptive retreatment indicated in a patient previously” for more details). Moreover, notwithstanding the excellent safety profile, in case of intravenous N-BP treatment with zoledronate or neridronate, we recommend the measurement of serum calcium, albumin, and, eventually, phosphate levels within 7 days after infusion to assess the possible occurrence of hypocalcemia and hypophosphatemia. In trials using zoledronate, mild, generally asymptomatic hypocalcemia (e.g., defined as ionized calcium below 1.21 mM) was reported in 2–6% of patients [73–76] and was more often described in patients with low vitamin D levels [94–96].

Severe and life-threatening hypophosphatemia has been more rarely described after intravenous zoledronic acid infusion [97–99]*.* Likewise, an assessment of serum creatinine and glomerular filtration rate is generally indicated before treatment and thereafter, in case of patient with mild renal impairment, due to the risk of nephropathy [100].

**Table:** 

Quality of evidence (GRADE)	Recommendation	Strength of recommendation
GRADE + +	In patients with PDB treated with N-BPs, we recommend assessing:	Strong (positive)
	Serum levels of t-ALP (or, alternatively, levels of P1NP, uNTx and b-ALP when recommended according to disease extension) at least once every year to evaluate the disease activity	Strong (positive)
	Serum levels of total calcium, albumin, and phosphate within 7 days after the intravenous infusion of the more potent N-BPs to assess the occurrence of electrolyte disorders	Weak (positive)
	Serum levels of calcium and t-ALP (or, alternatively, levels of P1NP, uNTx and b-ALP when recommended according to disease extension) when occurs a recurrence of pain at affected skeletal sites	Weak (positive)
	Serum levels of creatinine and the creatinine clearance at least within a year to estimate the occurrence of N-BP-related nephropathy	Serum levels of t-ALP (or, alternatively, levels of P1NP, uNTx and b-ALP when recommended according to disease extension) at least once every year to evaluate the disease activity

##### In a patient treated for PDB with persistent painful symptoms, is integration with analgesic therapy appropriate?

Assuming that specific RCTs regarding this question have not been published, in the clinical practice, integrative treatments with analgesics are often used in PDB, when antiresorptives are not able to achieve a satisfactory relieve of the painful symptoms. Indeed, in the PRISM study, most of the recruited patients reported the use of analgesics, non-steroidal anti-inflammatory agents (NSAIDs), and treatments for neuropathic pain at some point in the trial, in addition to N-BP therapy [81, 82]. In fact, very often in PDB bone pain can be mostly related to complications such as secondary osteoarthritis or neuropathy, rather than to active osteolytic lesions. In this respect, the integrative analgesic scheme can start with the “classic” paracetamol and move towards more powerful molecules, such as NSAIDs. However, nerve compression pain may improve better with anti-neuropathic agents such as amitriptyline, gabapentin, or pregabalin [50, 81, 101]. Absolute or relative contraindications to the use of the above-mentioned drug categories should be considered, and do not differ substantially from those established for the general, non-pagetic population, in relation to age, sex and presence of co-morbidities or any concomitant therapy. Importantly, all these procedures should not be regarded as an alternative to antiresorptive agents for the clinical management of PDB (since they do not suppress disease activity and progression at pagetic sites), but as adjunctive therapies for the control of pain [16]. Moreover, a lack of response or poor efficacy of analgesics, neuropathics and/or NSAIDs should always require an accurate screening (e.g., radiography, MRI or CT) to identify a different pathological mechanism underlying the painful symptoms (see point “Is a new radiological examination indicated in a previously treated PDB patient, in case exacerbation of painful symptoms at a pagetic site?”).

**Table:** 

Quality of evidence (GRADE)	Recommendation	Strength of recommendation
GRADE: +	We suggest the use of analgesics, NSAIDs and/or neuropathic agents as integrative treatments when antiresorptives are not able to achieve a satisfactory relieve of the painful symptoms	Weak (positive)

##### Is a new radiological examination indicated in a previously treated PDB patient, in case exacerbation of painful symptoms at a pagetic site?

The causes of pain at pagetic sites may be due to different mechanisms and conditions (e.g., osteolysis, osteoarthrosis, fracture, basilar invagination, spinal stenosis, or neoplastic degeneration) [2], which may require different therapeutic approaches, sometimes including orthopedic surgery. Thus, in the presence of exacerbation of painful symptoms at a pagetic site, we recommend performing a radiological analysis to identify the underlying cause. Fractures, and more frequently fissure fractures at weight bearing sites (particularly at the hip or tibia), are a common complication of PDB, often causing or worsening bone pain. An X-ray scan of the painful site can easily identify a single linear, cortical, fissure representing incomplete fracture on the convex surface of the long bone (Fig. 1C). Another common non-neoplastic complication in longstanding PDB that can be diagnosed by plain radiography is secondary osteoarthritis, frequently involving the hip and/or knee. However, particularly in case of skull and/or spine involvement, several neurologic complications may also occur (e.g., secondary to vertebral or calvarial enlargement) with resultant pain related to spinal and cranial nerve compression. In this case, CT and MRI are recommended for a better evaluation of the overall enlargement of bone and of the degree of spinal cord and/or cranial nerve encroachment. Radiographic signs of malignant degeneration of pagetic bone include aggressive osteolysis and cortical destruction, in absence of periosteal reaction. However, this latter feature makes sometimes difficult to differentiate sarcomatous transformation from a recrudescence of the lytic phase of PBD. A comparative analysis with previous radiographs may be of help to detect new osteolytic areas of sarcomatous degeneration. However, MRI or CT is also recommended to confirm the diagnosis. In particular, MRI can be a very useful procedure to identify the mass-like marrow replacement in the presence of a pagetic osteosarcoma [40]. An integrative assessment of t-ALP or other bone turnover markers (that are almost always increased in case of neoplastic degeneration) is also advised. A new radiological assessment is particularly indicated, if not mandatory, in case of recurrence of symptoms shortly after a therapeutic cycle with N-BPs. On the other side, irrespective of the presence of pain or other symptoms, a radiograph of the pagetic site(s) can be also performed 1 year after antiresorptive treatment to evaluate the refill of the osteolytic lesions [16].

**Table:** 

Quality of evidence (GRADE)	Recommendation	Strength of recommendation
GRADE: +	In case of exacerbation of painful symptoms in the pagetic site, it is recommended to carry out a targeted radiological assessment to exclude the presence of major complications such as fractures, osteoarthritis and above all neoplastic degeneration	Strong (positive)*

*Despite of the very limited evidence, the cost of radiological examination clearly out-weight the risk of misdiagnosing a fissure fracture or neoplastic degeneration.

##### Is antiresorptive retreatment indicated in a patient previously treated for PDB, with exacerbation of painful symptoms in the site of pagetic lesion?

There is very limited available information from RCTs about this issue. As first, the reliance on painful symptoms to indicate retreatment may be confused by the fact that not always these symptoms are the direct consequence of active PDB but may often be secondary to osteoarthritis or other complications not responding well to antiresorptive therapy [102, 103]. In the PRISM trial, the “symptomatic arm” received retreatment with BPs only if bone pain was present that was due to increased metabolic activity of PDB [81]. However, patients with pain were treated initially with analgesics or NSADs, while antiresorptive therapy was administered only if the response to these treatments was inadequate. Moreover, non-N-BPs or calcitonin was used as first choice antiresorptives and a limited number of patients received N-BPs (pamidronate or risedronate). With this approach, the prevalence of patients referring “pagetic” bone pain at 2 years decreased from 66 to 31%. Conversely, exacerbation of painful symptoms after PDB treatment with potent N-BPs (e.g., zoledronate) occurs less frequently. In the extension study of the core zoledronate RCT [75], which was performed in the subgroup of individuals reporting ALP normalization at the end of the trial, clinical relapse occurred in 9.2% of patients in the zoledronate group compared with 25.2% in the risedronate group. Among the overall group of 169 zoledronate-treated patients of the core RCT, only 6 meet retreatment criteria after 6–8 years of follow-up, of whom just 1 due to recurrence of bone pain [104]. Likewise, in a comparative trial of intramuscular and intravenous neridronate 2 of 27 (7.4%) and 4 of 29 patients (13.8%) in the intravenous and intramuscular groups, respectively, reported the recurrence or worsening of bone pain at 12 months and were treated with a second treatment course [105]. Of interest, all but one patient reported a decrease in bone pain after retreatment.

Thus, based on the above information and evidence deriving from the clinical practice, in the absence of more precise data, we suggest that a case-by-case assessment is required to establish the necessity of retreatment with N-BPs in PDB patients with recurrence of bone pain, in relation to the distance from the previous treatment regimen, the type of the used antiresorptive compound and, eventually, on the biochemical response. Importantly, in order to better characterize the cause of pain and support the decision, a radiological and biochemical screening is often necessary (see point “Is a new radiological examination indicated in a previously treated PDB patient, in case exacerbation of painful symptoms at a pagetic site?”). In fact, arthritic or neurogenic pain better responds to analgesics, or, if necessary, to narcotic therapy, and inadequate pain relief after these approaches might indicate the necessity of surgical intervention [102]. Moreover, the possibility of neoplastic degeneration must be ruled out.

Generally, the following clinical settings should be faced in case of the recurrence of bone pain:

1. In case of previous therapeutic cycle with calcitonin, non-N-BPs or oral N-BPs, we recommend retreatment with more potent intravenous N-BPs, such as zoledronate and neridronate, even at short distance, unless the pain is mainly attributable to osteoarthritis or neoplastic degeneration.
2. In case of previous intravenous cycle with zoledronate or neridronate, we recommend repeating the infusion starting from the following 12 months onwards (possibly favoring zoledronate).
3. In case pain recurrence occurs before 12 months from the previous intravenous cycle with zoledronate or neridronate, this is very often related to causes other than PDB activity, thus retreatment is generally not indicated.

**Table:** 

Quality of evidence (GRADE)	Recommendation	Strength of recommendation
GRADE: +	In case of recurrence or worsening of pain symptoms at the level of the pagetic lesions in patients previously treated with antiresorptive therapy, we suggest:	
	A new therapeutic course favoring intravenous N-BP regimens (zoledronate and neridronate) in case of previous treatment with calcitonin, non-N-BPs or oral N-BPs;	Weak (positive)
	When a previous therapeutic course with neridronate and zoledronate has been already performed, retreatment is advisable only if at least 12 months have elapsed. Otherwise, a further clinical-diagnostic evaluation is strongly recommended to exclude causes of pain unrelated to active PDB (e.g., osteoarthritis or neoplastic degeneration)	Weak (positive)

##### Is antiresorptive retreatment indicated in a previously treated PDB patient with increased total alkaline phosphatase (or other marker of bone turnover)?

Information about this issue has been mainly driven by the clinical practice and the only available, evidence-based data, derive from the PRISM study [81, 82], that has several limitations. In fact, most of the recruited patients had severe, long-lasting PDB, already treated with BPs and the use of potent N-BPs was not considered for most of the cases recruited in the “intensive” or “symptomatic” treatment arm (except in the subsequent extension study). However, outcomes from that trial did not support any benefit of a treatment strategy driven by the increase in bone turnover markers over treating PDB only in case of recurrence of bone pain.

Indeed, in most of the clinical trials with BPs (including N-BPs) and their extension studies, the increase in t-ALP above the normal reference range was considered an indication for retreatment [76, 104–106]. Thus, we have no evidence-based information deriving from leaving a PDB patient untreated over the long-term, in case of a new increase in t-ALP or other bone turnover markers. In this respect, however, some clues can be derived from the natural history of PDB and from the outcomes deriving from clinical trials with the less potent antiresorptive compounds [84, 87, 92, 107, 108]. In both cases, progression of osteolytic lesions and clinical worsening of PDB have been often described.

In the decision-making process, it is, therefore, essential to consider the potency and distance from previous treatment, in addition to the extent of the disease and the presence of complications. An increase in bone turnover shortly after the treatment course or the failure to normalize t-ALP and/or other markers during therapy with N-BPs should always prompt the execution of diagnostic tests, especially to exclude neoplastic degeneration or other causes of increased bone turnover other than PDB (e.g., primary hyperparathyroidism). If these latter are excluded, a new treatment course with potent intravenous N-BPs is suggested in case the patient has been treated with calcitonin, non-N-BPs or oral N-BPs. This should mainly include zoledronate (5 mg) or neridronate (200 mg), since a lower efficacy, including a sort of “resistance” to treatment, has been frequently described with intravenous pamidronate [76, 77, 105, 109–111]. Likewise, in the context of severe complications (e.g., paraplegia) or in the presence of lytic disease affecting long bones and weight bearing bones (which carries a high risk of deformity, osteoarthritis, and fractures), most experts suggest considering a new treatment course in case of a recurrence of disease activity, as suggested by an increase in bone turnover markers [16, 77, 94, 101–104, 111]. In a patient who had achieved normal t-ALP levels, the necessity of retreatment is generally considered once the levels have increased and exceed the upper normal limit by 25% or above [103, 111].

Importantly, with the wide use of potent N-BPs and considering the decrease in the cases with severe, polyostotic disease observed in the recent years, a new increase in bone turnover markers over the normal range is likely to become a rather rare occurrence, especially within 5–8 years from treatment [75–78, 104]. In these cases, obviously, a new therapeutic cycle with potent intravenous N-BPs can be considered.

**Table:** 

Quality of evidence (GRADE)	Recommendation	Strength of recommendation
GRADE +	In case of increase in t-ALP or any other bone turnover marker in patients previously treated for PDB:	
	A new therapeutic course favoring intravenous N-BP regimens (zoledronate and neridronate) is suggested in case of previous treatment with calcitonin, non-N-BPs or oral N-BPs;	Weak (positive)
	When a previous therapeutic course with neridronate and zoledronate has been already performed, retreatment is advisable only if at least 12 months have elapsed. Otherwise, a further clinical-diagnostic evaluation is recommended to exclude neoplastic degeneration or other causes of increased bone turnover other than active PDB	Weak (positive)

##### Is a therapeutic antiresorptive course indicated in a patient with PDB prior to orthopedic surgical procedure?

It is well known that skeletal sites affected by PDB have an increased vascularization [112]. It has, therefore, been suggested that such an increased vascularization could result in excessive blood loss, both in the case of fracture and, above all, in the case of orthopedic surgery in patients with PDB (whether related to osteosynthesis or prosthetics). Indeed, early studies with calcitonin and etidronate demonstrated a decrease in skeletal blood flow of pagetic skeletal sites after treatment, that correlated with the observed reductions in t-ALP [113, 114]. Based on this information, the Endocrine Society guidelines [16] and some consensus documents [103] recommended that the PDB patients should ideally be treated with BPs prior to elective orthopedic procedures in order to reduce intra-operative bleeding and, eventually, post-operative loosening of the prosthesis. However, there is no clear evidence from RCTs evaluating the effects of BPs versus placebo on blood loss during elective surgery in PDB. Some observational studies assessed the relationship between receiving anti-pagetic therapy and intra-operative blood loss following orthopedic surgery (e.g., hip or knee replacement, spinal surgery), with extremely discordant and hardly comparable findings, since different settings of patients were considered with different antiresorptive agents and a variable distance of treatment with respect to the various surgical procedures [115–119]. Diversity of materials used in the various procedures, in relation to the years in which these studies were conducted (from the end of the 70s to the end of the 90s), should be also considered. Thus, considering such a low-quality evidence, more recent guidelines concluded that there is not enough information to recommend the use of BPs prior to elective orthopedic surgery for PDB [17]. However, considering the cost-effectiveness and the safety of the potent intravenous N-BP regimens available to date, we suggest that a treatment course could be considered prior elective surgical procedure involving pagetic skeletal sites, at least in patients with active disease and/or naïve for antiresorptive treatment.

**Table:** 

Quality of evidence (GRADE)	Recommendation	Strength of recommendation
GRADE +	In a patient with PDB prior to elective surgical procedure, it is suggested, where possible and in the presence of active and/or previously untreated disease, to carry out a therapeutic course with intravenous N-BPs	Weak (positive)

##### Is antiresorptive treatment necessary in an “immobilized” PDB patient?

In the general population, long-term immobilization is known to result in deterioration of bone structure and substantial bone loss in both the trabecular and cortical compartments, which is generally characterized by relatively increased bone resorption and decreased bone formation [120, 121]. This may increase the risk of pathological fractures, hypercalcemia, hypercalciuria, and nephrolithiasis, which can be somewhat amplified by secondary or tertiary hyperparathyroidism due to vitamin D deficiency [122, 123]. Indeed, hypercalciuria following immobilization and the consequent increased risk of kidney stones have been included among the possible complications associated with PDB [2, 3, 124]. In a recent survey of Italian PDB cases, an increased prevalence of nephrolithiasis, even in the absence of immobilization, was described with respect to the general population, particularly in patients with polyostotic disease [125]. Likewise, hypovitaminosis D and hyperparathyroidism (including both primary and secondary forms) were also more prevalent in pagetic than in non-pagetic subjects [125, 126].

Based on these considerations, even in the lack of specific studies addressing the effects of immobilization on pagetic bone, we suggest that N-BP treatment can be prescribed to long-term immobilized patients with PDB, particularly in case of polyostotic disease and in the presence of hypercalcemia and/or increased bone turnover. The preventive correction of vitamin D deficiency and the maintenance of an adequate vitamin D status are also recommended (see in more detail point “Is supplementation with calcium and/or vitamin D appropriate in a PDB patient on antiresorptive treatment?”).

**Table:** 

Quality of evidence (GRADE)	Recommendation	Strength of recommendation
GRADE: +	We suggest N-BP treatment in long-term immobilized PDB patients (particularly in case of polyostotic disease and in the presence of hypercalcemia and/or increased bone turnover). A preventive correction of inadequate vitamin D status is mandatory before treatment	Weak (positive)

#### How to treat?

##### In a patient with newly diagnosed PDB requiring medical treatment, which antiresorptive agent should be preferred?

N-BPs represent the treatment of choice for PDB, given their superiority to calcitonin or non-N-BPs [70, 72, 92, 127, 128]. Despite a limited number of comparative studies with N-BPs have been performed in PDB, it is well established that zoledronate represents the most effective regimen, improving pain and allowing the long-term suppression of bone turnover for more than 5 years in most patients [72–78]. Together with its increased efficacy, zoledronate has been also considered a cost-effective approach for PDB [129, 130]. Thus, in the absence of contraindications or unless intravenous treatment cannot be performed, this drug should be considered as a first-line treatment option in most patients with PDB, and particularly in those with polyostotic disease or carriers of mutations in *SQSTM1*, *ZNF687* or *PFN1* genes, which generally have a more severe disease [53–57]. As an alternative, and in case of drug availability, intravenous neridronate should be preferred with respect to pamidronate, as indicated by a comparative trial [70, 76, 77]. In this case, however, the efficacy of neridronate over the long term appears lower than that described with zoledronate [76].

Moreover, either oral N-BPs or intramuscular neridronate can be considered as effective alternatives in those patients unable or unwilling to perform intravenous infusion [70, 92, 105, 127]. Conversely, the use of calcitonin and non-N-BPs is not recommended as a first treatment option for PDB. However, calcitonin may be considered for short-term management of PDB, in case BPs are contraindicated [17].

While oral N-BPs have been generally associated with mild to moderate gastrointestinal side effects (mainly esophageal irritation and upper gastrointestinal discomfort) sometimes impairing treatment compliance, intravenous N-BP infusion may cause the so-called “acute phase reaction”, mainly characterized by fever, musculoskeletal pain, arthralgia, and other flu-like symptoms. This adverse event is generally mild to moderate in severity and most symptoms generally resolve within 3–7 days after infusion. In a series of PDB cases treated with different intravenous N-BPs, acute phase reaction occurred in up to 50% of naïve PDB patients after the first exposure to the drug, or less frequently (below 15–20%) with subsequent N-BP infusions [97]. Conversely, concerning gastrointestinal side effects, a Cochrane review did not report any significant difference between PDB patients under oral BP treatment and placebo [72]. Moreover, the long-term use of N-BPs has been associated with rare, but severe, adverse events such as atypical femoral fractures and jaw osteonecrosis [2]. Both these adverse events occur less frequently in PDB than in patients treated with N-BPs for osteoporosis, likely due to the use of intermittent and shorter term treatment courses [70, 72]. Finally, N-BP treatment is contraindicated in patients with severe renal impairment (glomerular filtration rate < 35 mL/min) [16], so that in this case, calcitonin or denosumab, could be used (albeit information about denosumab use in PDB only derives from few isolate case reports) [17].

**Table:** 

Quality of evidence (GRADE)	Recommendation	Strength of recommendation
GRADE: + +	In a patient with newly diagnosed PDB requiring medical treatment, the use of N-BPs is recommended as first-line therapy. In patients with symptomatic and/or polyostotic PDB or carriers of known mutations (SQSTM1, ZNF687, PFN1), zoledronate should be preferred as the first-line and cost-effective treatment option	Strong (positive)

##### Is supplementation with calcium and/or vitamin D appropriate in a PDB patient on antiresorptive treatment?

Several studies, including recent surveys in large patient cohorts, demonstrated that vitamin D deficiency (25OHD levels < 20 ng/ml) is frequent in PDB [126, 131, 132]. As previously outlined, N-BP treatment (particularly in case of potent intravenous compounds) may result in hypocalcemia that is more common in case of vitamin D deficiency. Indeed, albeit a limited number of patients experienced hypocalcemia in the registrative zoledronate trial [73], in this as well as in most of the N-BP trials for PDB all patients received calcium and vitamin D supplementation. Recent data demonstrate that cholecalciferol treatment, at the dosage proposed in the general population to reach and/or maintain normal 25OHD levels, is effective and safe for the correction of vitamin D deficiency in patients with PDB [126], and also reduces the risk of acute phase reaction after intravenous N-BP treatment [94]. Thus, based on the above information, we recommend correcting hypovitaminosis D according to the indications recently provided by the SIOMMMS position paper [133], and that all PDB patients on antiresorptive treatment receive adequate calcium and vitamin D supplementation. In this respect, an assessment of 25OHD levels before N-BP prescription is also advised. It is interesting to point out that combined N-BP and cholecalciferol treatment may also have extraskeletal benefits, reducing circulating glucose and atherogenic lipids, as recently observed in a study of patients with metabolic bone disorders, including PDB [134].

**Table:** 

Quality of evidence (GRADE)	Recommendation	Strength of recommendation
GRADE: + +	In patients with PDB, we recommend measuring 25OHD levels and correct the condition of hypovitaminosis D according to the indications recently provided by the SIOMMMS	Strong (positive)
	During treatment with N-BPs, it is advisable to ensure an adequate calcium and vitamin D intake	

##### Is it appropriate to change the therapeutic antiresorptive regimen in a previously treated PDB patient who is experiencing relapse of the disease (symptomatologic and/or biochemical)?

A sort of “resistance” to treatment with repeated treatment courses of the same antiresorptive compound has been described in some patients with PDB [110]. This occurs more frequently with calcitonin and non-N-BPs but has been also described after repeated intravenous courses of pamidronate [76, 105, 106, 109, 110]. As suggested by a limited number of comparative trials [92, 127] and more recently outlined by a Cochrane review [72], N-BPs are more effective than non-N-BPs both on the normalization of bone turnover markers and the reduction of bone pain. In recent comparative clinical trials between N-BPs, either zoledronate and/or neridronate demonstrated an increased efficacy in the control of bone pain with respect to intravenous pamidronate [76] or oral N-BPs such as risedronate [73–75]. Moreover, PDB recurrence is less frequent and occurs later in case of intravenous N-BP treatment with neridronate and particularly with zoledronate, which appears effective over the long term in most patients. Thus, based on these considerations, in case of disease recurrence (e.g., due to worsening of bone pain and/or increase in bone turnover markers), we recommend a new treatment course with N-BPs, favoring the more effective intravenous compounds such as zoledronate, or eventually neridronate.

As outlined in the previous sections, caution should be taken when recurrence occurs shortly after N-BP treatment, as this is usually related to other clinical causes than active PDB.

**Table:** 

Quality of evidence (GRADE)	Recommendation	Strength of recommendation
GRADE: + +	In a PDB patient with disease relapse, in the necessity of a new treatment course, we recommend favoring intravenous regimens with zoledronate and, eventually, neridronate (which have shown greater long-term efficacy over other compounds)	Strong (positive)

## Summary and conclusions

PDB is a focal disorder of bone metabolism that is becoming less frequent and is often overlooked in clinical practice, so that the diagnosis is made at a later stage. Although the prevalence of monostotic and asymptomatic PDB cases is increasing, the progression of the disease can lead to invalidating complications that compromise the quality of life. Based on these considerations, the somewhat contrasting outcomes from previous guidelines [16, 17] and the results of more recent studies, the SIOMMMS found the necessity to provide precise and up to date indications for the diagnosis and treatment of the disease. In the lack of good evidence from RCT to support clear recommendations, available information from the literature together with expert opinion of the panel was used to provide suggestions for the clinical practice.

As summarized in Fig. 1, the diagnosis of PDB should be mainly based on the presence of symptoms, when present, together with the typical biochemical and radiological features. A bone scan (99mTc-MDP) is also recommended to assess disease extension or detect early pagetic lesions (e.g., in subjects with family history of PDB and/or carriers of mutations associated with the disorder). Less clear evidence is available regarding treatment indications. While there were no doubts to recommend N-BPs treatment to symptomatic PDB cases at diagnosis, some debate arose concerning the necessity of treatment in patients without symptoms as well as in previously treated patients in the presence of biochemical recurrence (e.g., an increase in t-ALP or other bone turnover markers). However, in view of safety-efficacy profile of N-BPs and the long-lasting effects of single treatment course with intravenous compounds (i.e., zoledronate), a suggestion to treat most if not all PDB cases with N-BPs at the time of diagnosis was released. We hope that future research can provide more clear indications about this and other conflicting issues.
